# Supplementary material for: Function-based high-throughput screening for antibody antagonists and agonists against G protein-coupled receptors
Source: Commun Biol. 2020 Mar 26;3:146. doi: 10.1038/s42003-020-0867-7 (PMC7099005; doi:10.1038/s42003-020-0867-7)
Supplement: Supplementary file 1 — Supplementary Information [file 42003_2020_867_MOESM1_ESM.pdf]

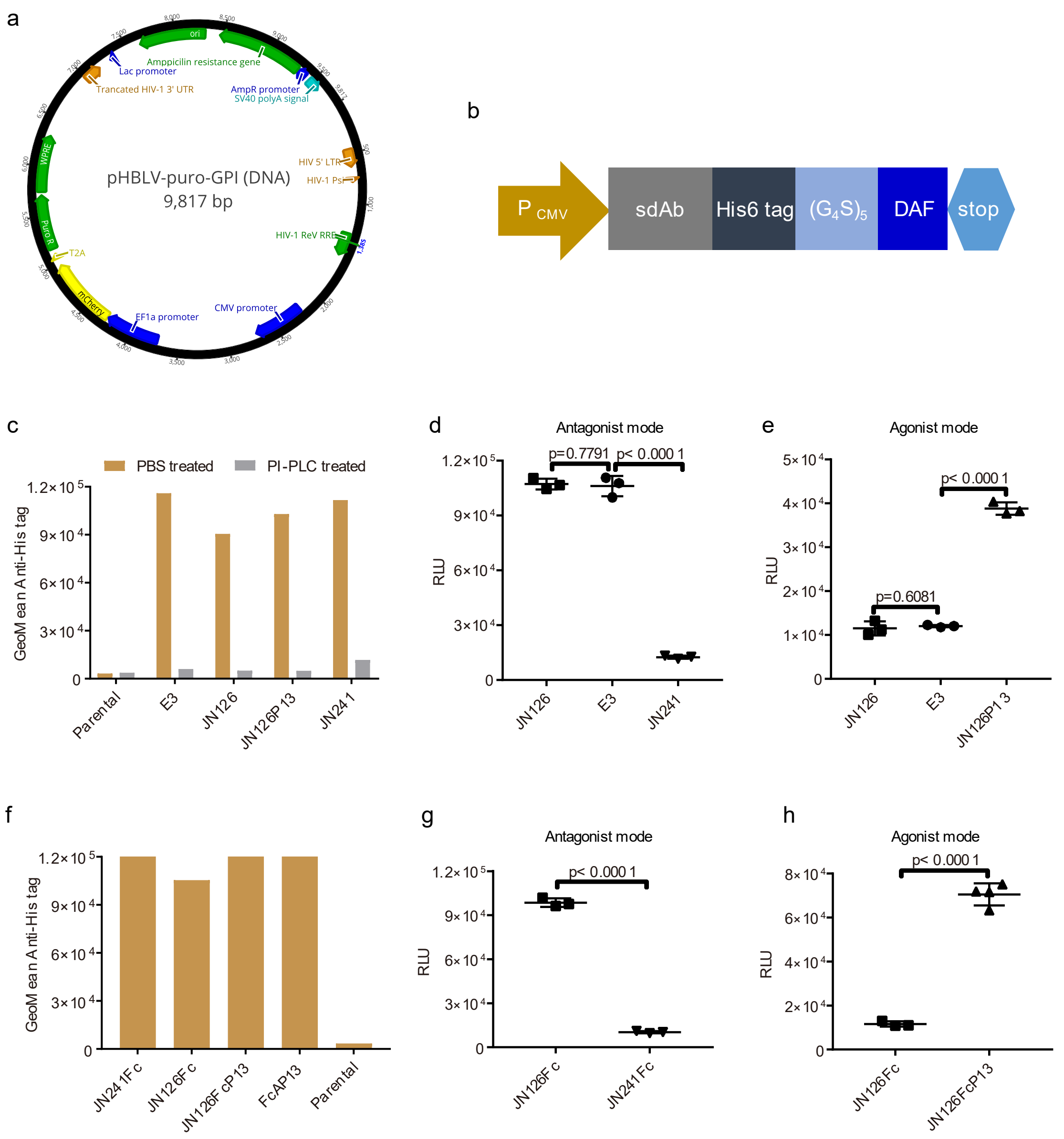

Supplementary Figure 1 Schemtic diagram of the lentiviral vector and POC study in CHO-k1/huAPJ PathHunter β-arrestin reporter assay cell line. **a-b.** Schematic diagram of the lentiviral vector pHBLV-puro-GPI (**a**) and its expresion cassette (**b**). His6 tag: A six histidine tag; $(G_4S)_5$ linker: Five repeats of G_4S linker; DAF: The C-terminal 34 amino acid residues of decay accelerating factor. **c.** Flow cytometry of recombinant lentivirus-transduced CHO-k1/APJ β-arrestin assay cells with or without PI-PLC treatment. **d-e.** β-arrestin assay at antagonist **(d)** or agonist **(e)** mode with GPI-

anchored sdAbs E3, JN126, and JN241, and JN126P13 fusion. **f.** Flow cytometry of CHO-k1/APJ β-arrestin assay cells transduced with recombinant lentiviruses containing the genes encoding sdAb and/or ligand and Fc fusions. **g-h.** β-arrestin assay at antagonist **(g)** or agonist **(h)** mode with GPI-anchored sdAb and/or ligand and Fc fusions, JN126Fc, JN241Fc and JN126P13Fc fusions. Data was expressed as mean with SD in (**d-e)** and (**g-h)**. Statistical analysis was done using unpaired two-tailed T test.

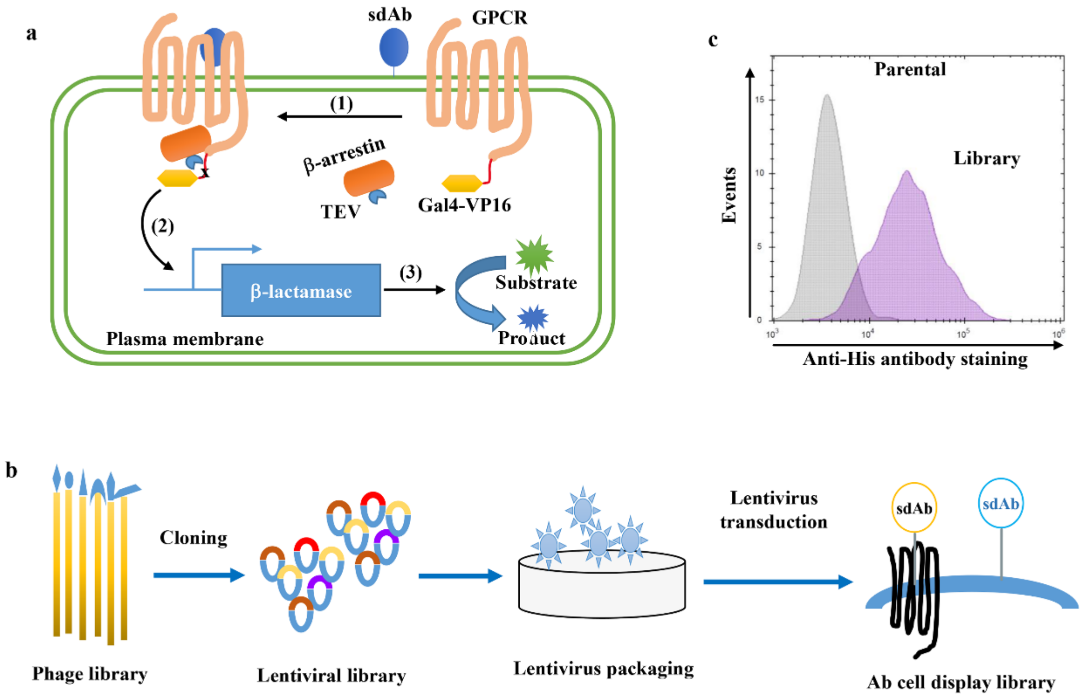

Supplementary Figure 2 Schematics of the GPI-anchored sdAb library construction and mechanism of Tango assay system. a. Mechanism of Tango/APJ β-arrestin assay. **b.** Schematic illustration of the workflow for generation of GPI-anchored sdAb library. **c.** Flow cytometry of GPI-anchored sdAb library by staining with anti-his antibody.

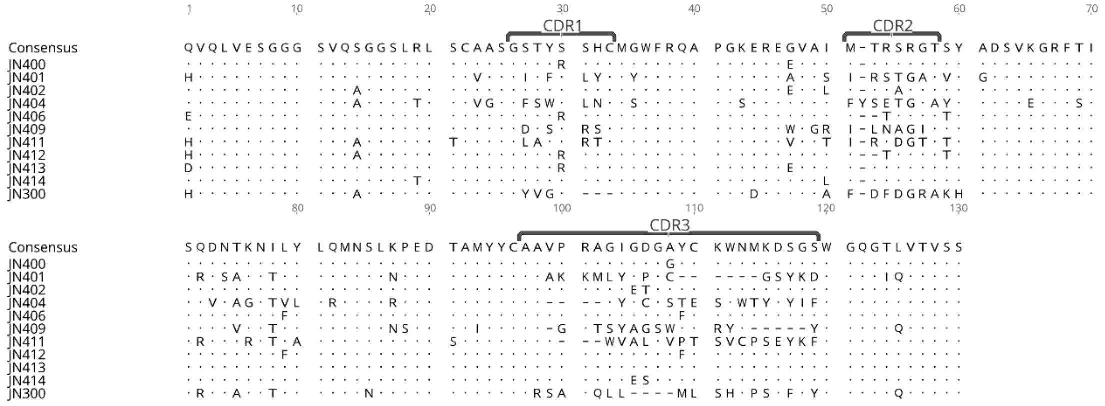

Supplementary Figure 3 Amino acid sequence alignment of isolated functional sdAbs against human APJ. Complementarity determining region (CDR) 1, 2 and 3 (CDR1, CDR2 and CDR3) are indicated. ".": the same amino acid residue as that in the consensus sequence at the same position. "-": lack of any amino acids at this position.

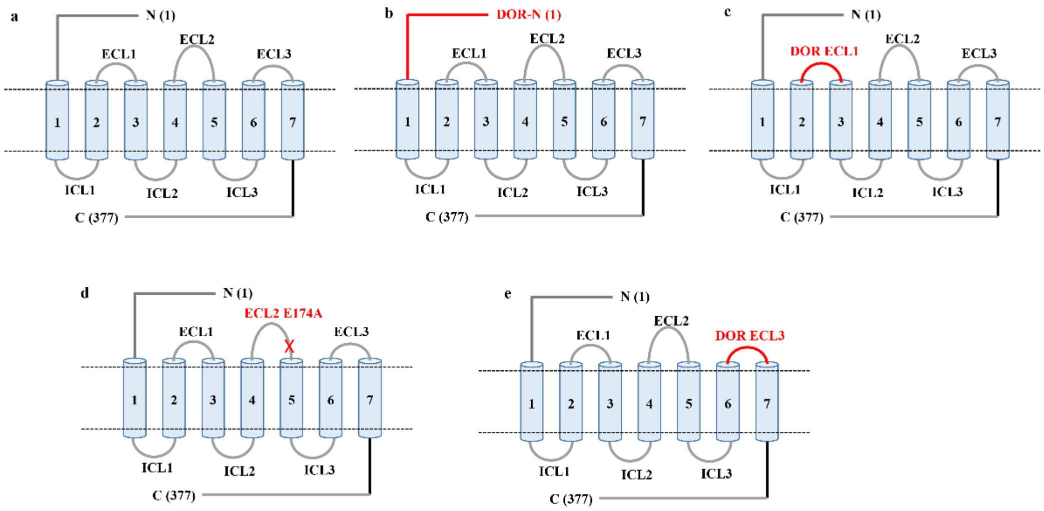

Supplementary Figure 4 Schematic diagrams of APJ/DOR domain-swapped mutants for epitope localization of isolated APJ functional sdAbs. a. WT APJ. **b**. APJ/DOR N-terminus-swapped mutant. **c**. APJ/DOR ECL1-swapped mutant. **d**. WT APJ E174A site mutant. **e**. APJ/DOR ECL3-swapped mutant. Swapped domains and mutation site in the ECL2 are highlighted in red.

Supplementary Table 1 Sequence analysis and expression profile of the functional APJ sdAbs. Sequence analysis was done using IMGT/V-QUEST software against alpaca reference sequences. All expressions were done in FreeStyle 293 expression system.

**Name**	**Function**	**Germline family**			**Length (AA)**			**Yield (mg/L culture)**
		V	D	J	CDR1	CDR2	CDR3	
JN400	Antagonist	IGHV3S53*01	IGHD7*01	IGHJ6*01	8	7	23	26.00
JN401	Antagonist	IGHV3S53*01	IGHD5*01	IGHJ4*01	8	7	17	10.00
JN402	Antagonist	IGHV3S53*01	IGHD2*01	IGHJ3*01	8	7	23	26.50
JN404	Antagonist	IGHV3S1*01	IGHD6*01	IGHJ3*01	8	8	19	2.92
JN406	Antagonist	IGHV3S53*01	IGHD1*01	IGHJ3*01	8	6	23	27.25
JN409	Antagonist	IGHV3S53*01	IGHD6*01	IGHJ4*01	8	7	17	28.67
JN411	Antagonist	IGHV3S55*01	IGHD7*01	IGHJ3*01	8	7	20	27.67
JN412	Antagonist	IGHV3S53*01	IGHD1*01	IGHJ3*01	8	6	23	38.47
JN413	Antagonist	IGHV3S53*01	IGHD2*01	IGHJ6*01	8	7	23	34.30
JN414	Antagonist	IGHV3S53*01	IGHD2*01	IGHJ3*01	8	7	23	23.27
JN300	Agonist	IGHV3S53*01	IGHD3*01	IGHJ6*01	5	7	19	2.44
