# Supplementary material for: Function-based high-throughput screening for antibody antagonists and agonists against G protein-coupled receptors
Source: Commun Biol. 2020 Mar 26;3:146. doi: 10.1038/s42003-020-0867-7 (PMC7099005; doi:10.1038/s42003-020-0867-7)
Supplement: Supplementary file 2 — Description of Additional Supplementary Files [file 42003_2020_867_MOESM2_ESM.pdf]

File Name: Supplementary Data
Description: Data underlined in the graphs and charts presented in the figure 1-5 in the main text
