# Supplementary material for: Function-based high-throughput screening for antibody antagonists and agonists against G protein-coupled receptors
Source: Commun Biol. 2020 Mar 26;3:146. doi: 10.1038/s42003-020-0867-7 (PMC7099005; doi:10.1038/s42003-020-0867-7)
Supplement: Supplementary file 4 — Reporting Summary [file 42003_2020_867_MOESM4_ESM.pdf]

nature research

Corresponding author(s): Mei-Yun Zhang

Last updated by author(s): 2/22/2020

Reporting Summary

Nature Research wishes to improve the reproducibility of the work that we publish. This form provides structure for consistency and transparency in reporting. For further information on Nature Research policies, see Authors & Referees and the Editorial Policy Checklist.

Statistics

For all statistical analyses, confirm that the following items are present in the figure legend, table legend, main text, or Methods section.

n/a	Confirmed	
☐	☒	The exact sample size (n) for each experimental group/condition, given as a discrete number and unit of measurement
☐	☒	A statement on whether measurements were taken from distinct samples or whether the same sample was measured repeatedly
☐	☒	The statistical test(s) used AND whether they are one- or two-sided<br>*Only common tests should be described solely by name; describe more complex techniques in the Methods section.*
☒	☐	A description of all covariates tested
☒	☐	A description of any assumptions or corrections, such as tests of normality and adjustment for multiple comparisons
☐	☒	A full description of the statistical parameters including central tendency (e.g. means) or other basic estimates (e.g. regression coefficient) AND variation (e.g. standard deviation) or associated estimates of uncertainty (e.g. confidence intervals)
☐	☒	For null hypothesis testing, the test statistic (e.g. F, t, r) with confidence intervals, effect sizes, degrees of freedom and P value noted<br>*Give P values as exact values whenever suitable.*
☒	☐	For Bayesian analysis, information on the choice of priors and Markov chain Monte Carlo settings
☒	☐	For hierarchical and complex designs, identification of the appropriate level for tests and full reporting of outcomes
☒	☐	Estimates of effect sizes (e.g. Cohen's d, Pearson's r), indicating how they were calculated

Our web collection on statistics for biologists contains articles on many of the points above.

Software and code

Policy information about availability of computer code

Data collection	BD FACS software, ForeCyt Enterprise Client Edition 5.1 (R1) version 5.4.6180, Envision, ClarioStar, LEICA DMi8
Data analysis	GraphPad Prism 7, Flowjo_V10, ForeCyt Enterprise Client Edition 5.1 (R1) version 5.4.6180

For manuscripts utilizing custom algorithms or software that are central to the research but not yet described in published literature, software must be made available to editors/reviewers. We strongly encourage code deposition in a community repository (e.g. GitHub). See the Nature Research guidelines for submitting code & software for further information.

Data

Policy information about availability of data

All manuscripts must include a data availability statement. This statement should provide the following information, where applicable:

- Accession codes, unique identifiers, or web links for publicly available datasets
- A list of figures that have associated raw data
- A description of any restrictions on data availability

Fig. 1 POC study in U2OS/APJ b-arrestin reporter cell line and the construction of a GPI-anchored sdAb library. Fig. 2 Function-based cell sorting and screening for sdAb antagonists. Fig. 3 Characterization of isolated sdAb antagonists for function and epitopes. Fig. 4 Function-based cell sorting and screening for agonistic cell clones. Fig. 5 Characterization of sdAb agonist JN300 for function and epitope.

Field-specific reporting

Please select the one below that is the best fit for your research. If you are not sure, read the appropriate sections before making your selection.

☒ Life sciences ☐ Behavioural & social sciences ☐ Ecological, evolutionary & environmental sciences

For a reference copy of the document with all sections, see nature.com/documents/nr-reporting-summary-flat.pdf

Life sciences study design

All studies must disclose on these points even when the disclosure is negative.

Sample size	9 samples for correlation study
Data exclusions	1 sample was excluded in the correlation study due to lack of measurable IC50.
Replication	Each experiment was repeated twice. Each sample was tested in duplicates or in triplicates in functional assays. Epitope localization was carried out three times, but each sample was not duplicated.
Randomization	na
Blinding	na

Reporting for specific materials, systems and methods

We require information from authors about some types of materials, experimental systems and methods used in many studies. Here, indicate whether each material, system or method listed is relevant to your study. If you are not sure if a list item applies to your research, read the appropriate section before selecting a response.

Materials & experimental systems

n/a	Involved in the study	
☐	☒	Antibodies
☐	☒	Eukaryotic cell lines
☒	☐	Palaeontology
☒	☐	Animals and other organisms
☒	☐	Human research participants
☒	☐	Clinical data

Methods

n/a	Involved in the study	
☒	☐	ChIP-seq
☐	☒	Flow cytometry
☒	☐	MRI-based neuroimaging

Antibodies

Antibodies used	Anti-his6 antibody (Genescript, A00186, Lot #17K001367), Alexa488 conjugated goat anti-mouse IgG (H+L) antibodies (Invitrogen, A11029, Lot #1942237, #2066710) , Alexa488 conjugated mouse anti-HA Clone 16B12 (Invitrogen, A21287, Lot #1971419).
Validation	positive and negative controls were included in the experiments.

Eukaryotic cell lines

Policy information about cell lines

Cell line source(s)	Invitrogen (293FT, 293F, Tango/APJ beta-arrestin), DiscoverX (CHO-APJ/beta-arrestin, U2OS/APJ)
Authentication	na
Mycoplasma contamination	Cell lines were purchased from Invitrogen and DiscoverX with no macroplama contamination.
Commonly misidentified lines (See ICLAC register)	na

Flow Cytometry

Plots

Confirm that:

- [x] The axis labels state the marker and fluorochrome used (e.g. CD4-FITC).
- [x] The axis scales are clearly visible. Include numbers along axes only for bottom left plot of group (a 'group' is an analysis of identical markers).
- [x] All plots are contour plots with outliers or pseudocolor plots.
- [x] A numerical value for number of cells or percentage (with statistics) is provided.

Methodology

Sample preparation	trypsin digestion, washing with 1 X PBS, staining with ligand or antibodies or reporter enzyme substrate
Instrument	FACSJazz (BD), IntelliCyt iQue Screener (Sartorius)
Software	BD FACS Software, Flowjo, ForeCyt Enterprise Client Edition 5.1
Cell population abundance	sorted cell population ranges from 5-10%.
Gating strategy	set initial cell population by using FSC/SSC, set sorting cell population by excluding cell aggregates and with desired florescent readouts.

- [x] Tick this box to confirm that a figure exemplifying the gating strategy is provided in the Supplementary Information.
